# Dual method use for protection of pregnancy and disease prevention among HIV-infected women in South East Nigeria

**DOI:** 10.1186/1472-6874-14-39

**Published:** 2014-03-07

**Authors:** Lucky O Lawani, Azubuike K Onyebuchi, Chukwuemeka A Iyoke

**Affiliations:** 1 School of Postgraduate Studies, Department of Community Medicine, University of Nigeria, Enugu Campus, Enugu State, Nigeria; 2 Department of Obstetrics & Gynaecology, Federal Teaching Hospital, Abakaliki, Ebonyi State, Nigeria; 3 Department of Obstetrics & Gynaecology, University of Nigeria Teaching Hospital, Ituku-Ozalla, Enugu State, Nigeria

**Keywords:** HIV, AIDS, Dual methods, Safer sex, Parturient, Barrier, Safer sex

## Abstract

**Background:**

sub-Saharan Africa continue to bear the greatest burden of HIV/AIDS epidemic due to its large population, high fertility rate and unmet contraceptive need, most especially with poor uptake of dual methods (use of condom and another effective family planning method) which protects against STIs/HIV and unplanned pregnancy. The aim of this study was to assess the awareness, pattern and practice of dual methods by HIV infected women, and factors influencing its use in southeast Nigeria.

**Methods:**

This was a cross sectional descriptive study of 658 HIV positive women attending the PMTCT/postnatal/family planning clinics in three health facilities in southeast Nigeria. An interviewer administered semi-structured questionnaire was used to abstract needed information. The data were analyzed with Epi-info™ version 7.0 (Centers for Disease Control and Prevention, Atlanta, GA, USA), Odd ratio was determined and the test of statistical significance was with Fisher exact test at 95% CI.

**Results:**

The mean age of the participants was 29 ± 4.3 years. All the respondents were aware of their HIV status, 62.4% did not know their partners status; 23.1% were sero-concordant, while 14.5% were sero-discordant. Most (67.9%) of the respondents lack awareness on dual methods with only 179/658 (27.2%) practicing it. The commonest (141/179; 78.9%) dual method used was a combination of condom and injectable hormonal contraceptives. Lack of awareness (222/479; 46.3%) and non disclosure (133/479; 27.8%) were the main reasons for non use of dual method in the present study. STI’s was higher amongst non users with odd ratio of 1.74 (1.26-2.41), p-value < 0.0004. Unplanned pregnancy was higher in non users with odd ratio of 3.89 (2.52-6.00), p-value < 0.0000 at 95% CI.

**Conclusions:**

The awareness and uptake of dual methods amongst HIV infected women in southeast Nigeria is still low and thus associated with a higher risk of STIs and unplanned pregnancy. It is expected that increased awareness, uptake and consistent use will help prevention new infections of HIV/STIs and unplanned pregnancy.

## Background

The human immunodeficiency virus infection (HIV) and acquired immunodeficiency syndrome (AIDS) pandemic is one of the most serious crisis the world is facing today [1-4]. While new cases have been reported in all regions of the world, 95% of new infections occur in individuals who resides in low-and middle-income countries, particularly in sub-Saharan Africa [5], which continues to bear the greatest burden of the HIV and AIDS epidemic due to its high fertility rate and large population, accounting for 67.6% of the 35 million people living with HIV worldwide, 69.2% of the 2.6 million total new infections and 72.2% of the 1.8 million deaths worldwide in 2009 [1].

In Africa, HIV infected women just like those who are un-infected have unmet contraceptive needs for birth control [6]. They also require protection against STI’s (sexually transmitted infections) with barrier methods for safer sex. International and national recommendations are that HIV infected women should use dual methods; a barrier method like condom for safer sex against sexually transmitted infections, concurrently with another effective family planning method in those who desire contraception post partum and in the preconception period to ensure all future pregnancies are desired and planned [1,2]. Studies have shown that HIV sero-discordant and sero-concordant couples, who continue to practice unprotected sex, will increase their exposure to mutant or multiple strains of the human immunodeficiency virus, as well as other sexually transmitted infections (STIs) and unplanned pregnancy, if the use of dual method for disease and pregnancy prevention was not practiced [4-9]. The problems and risks of unprotected sex such as unintended pregnancy and sexually transmitted infections are inextricably linked [10], thus the recommendation that all HIV positive individuals should practice safer sex using dual methods as protection against STIs and unplanned pregnancy [1]. This recommendation is a potential way of reducing the scourge and spread of sexually transmitted diseases including HIV/AIDS and also improving the reproductive health challenges associated with unplanned pregnancy in the HIV infected.

It is against this background that this study was designed to assess the awareness, pattern and practice of dual method by HIV infected women, as well as factors influencing its use in southeast Nigeria with the aim of making recommendations on how to further improve the sexual and reproductive health services offered to HIV infected women and their sexual partners.

## Methods

This was cross sectional descriptive questionnaire based study conducted over a one year period (1^st^ June, 2012 to 30^th^ May, 2013) in three major health facilities (Federal Teaching Hospital, Mile Four Clinic and General Hospital Onueke) offering PMTCT/postnatal and family planning services to women infected with HIV/AIDS in Ebonyi state, Southeast Nigeria. Ethical clearance was obtained from the ethics and research committees of the institutions. Parturients in these institutions were seen at the PMTCT/post natal clinic and family planning clinic six weeks post partum; for post natal care and family planning counseling and there after offered contraceptive methods of their choice, for those who desire early contraception. Those who were HIV infected also received PMTCT services as well.

All HIV positive parturients who had delivered and attending these clinics for six weeks postnatal care, who gave consent to be part of the research were recruited for the study. An interviewer administered semi-structure questionnaire was used to obtain information on the socio-demographic characteristics, the pattern and practice of dual methods and factors influencing its use before conception. Information on concurrent and consistent use of dual (two) methods were also sought. Dual method was defined as the use of two methods; made up a barrier method in combination with another effective family planning method as recommended by the World Health Organization and the Nigerian national PMTCT guideline [1,2]. Sexually transmitted infections was defined as infections that were primarily contacted through person-to-person sexual contact, while the HIV infected was defined as those with confirmed HIV positive test results. A total of 658 women participated in the study; all the questionnaires were suitable for analysis. The data were analyzed with Epi-info™ version 7.0 (Centers for Disease Control and Prevention, Atlanta, GA, USA) and the process involved determination of mean, percentages and inferential statistics to determine Odd ratio (OR) at 95% Confidence interval (CI). Test of statistical significance was with Fisher exact test with p value < 0.05 considered as statistically significant.

## Results

The socio demographic characteristics of the participants (Table 1), indicates a relatively young population within the reproductive age group. The mean age of the participants was 29 ± 4.3 years. Majority (92.5%) of who were heterosexuals, 7.5%; bisexuals, and there were no homosexuals. Table 2 shows that most of the respondents were aware of their HIV status for a period of at least 1-5 years, it also indicate that 411/658 (62.4%) of the participants did not know their partners HIV status, while those that were aware of their partner’s status were either sero-concordant (23.1%) or sero-discordant (14.5%). Most (67.9%) of the respondents lack awareness on dual methods as a form of safer sex practice and birth control, while 94.1% of those who were aware got informed during visits to health care facilities.

**Table 1:** Socio demographic characteristics of participants, N = 658

**Variables**	**n (%)**
**Age (years)**	
<20	5 (0.7)
21-25	141 (21.5)
26-30	250 (38.0)
31-35	165 (25.0)
36-40	95 (14.5)
>40	2 (0.3)
**Marital status**	
Married	598 (90.9)
Single	27 (4.1)
Separated	30 (4.5)
Divorced	3 (0.5)
**Educational status**	
None	85 (13.0)
Primary	232 (35.2)
Secondary	305 (46.3)
Tertiary	36 (5.5)
**Employment status**	
Employed	442 (67.2)
Unemployed	216 (32.8)
**Residence**	
Urban	251 (38.2)
Rural	407 (61.8)
**Parity**	
<1	240 (36.5)
1-4	411 (62.4)
≥5	7 (1.1)

**Table 2:** Duration of HIV infection, partners’ HIV status, awareness of dual methods and source of information

**Variables**	**n (%)**
**Duration of infection/diagnosis, N = 658**	
<1 year	165 (25.0)
1-5 years	449 (68.3)
>5 years	44 (6.7)
**Partners’ HIV status, N = 658**	
Unknown	411 (62.4)
Positive (sero-concordant)	152 (23.1)
Negative (sero-discordant)	95 (14.5)
**Awareness, N = 658**	
Unaware	447 (67.9)
Aware	211 (32.1)
**Source of information, N = 211**	
Health care facility	199 (94.1)
Mass media	6 (3.0)
Books/magazines	3 (1.3)
Peers	2 (1.1)
School	1 (0.5)

Table 3 shows that only 179/658 (27.2%) of respondents practiced dual method of contraception, indicating a low or poor uptake; the commonest (141/179; 78.9%) form of dual method used was a combination of condom and injectable hormonal contraceptives, while the least (2/179; 1.3%) form used was a combination of condom and intra uterine contraceptive device (IUCD). A proportion (12.2%) of the participants had multiple sexual relationships in the past one year preceding the survey, while 87.8% did not. Of those who engaged in multiple sexual relationships, only 17.4% practiced dual method, 52.6% used only condom, while 30.0% did not used any form of contraception or safer sex method. Dual method was used consistently in the preconception period by 26.8%, while 73.2% used it sometimes (non-consistently) as shown in Table 4. Non users of dual method gave lack of awareness of the practice (222/479; 46.3%) and non disclosure (133/479; 27.8%) of their HIV status as the main reasons for non use. The other reasons are presented in Table 4.

**Table 3:** Practice of dual methods and types used

**Variables**	**n (%)**
**Practice, N = 658**	
No	479 (72.8)
Yes	179 (27.2)
**Types used, N = 179**	
Condom and hormonal injectables	141 (78.9)
Condom and oral pills	33 (18.4)
Condom and implants	3 (1.4)
Condom and IUCD	2 (1.3)

IUCD = Intra uterine contraceptive device.

**Table 4:** Pattern of use of dual methods and reasons for non-use

**Variables**	**n (%)**
**Pattern of use, N = 179**	
Sometimes	131 (73.2)
Consistently	48 (26.8)
**Reasons for non-use, N = 479**	
Lack of awareness	222 (46.3)
Non-disclosure	133 (27.8)
Decline by partner	70 (14.6)
Non-availability	20 (4.1)
Religious belief	13 (2.7)
Cost	10 (2.2)
Culturally unaccepted	6 (1.3)
Inhibits sexual satisfaction	5 (1.0)

Sexually transmitted infections (STIs) were higher amongst non users than users of dual method with odd ratio of 1.74 (1.26-2.41) at 95% CI, p- value < 0.0004 using Fisher Exact Test which was statistically significant (Table 5). Table 5 also shows that unplanned pregnancy was higher in non users than users with odd ratio of 3.89 (2.52-6.00) at 95% CI, p-value < 0.0000 using Fisher Exact test, this was also statistically significant.

**Table 5:** Comparison of incidence of STI and unplanned pregnancy in users (N = 179) and non-users (479) of dual methods

**Variables**	**n (%)**	**OR**	**CI**	**p-value**
**STI (non-users)**				
Yes	204 (42.5)			
No	275 (57.5)			
**STI (Users)**				
Yes	0 (0.0)			
No	179 (100)			
		1.74	1.26-2.41	*0.0004
**Unplanned pregnancy (non-users)**				
Yes	356 (74.3)			
No	123 (25.7)			
**Unplanned pregnancy (users)**				
Yes	0 (0.0)			
No	179 (100)			
		3.89	2.52-6.00	*0.0000

*statistically significant.

OR = Odd ratio.

CI = Confidence interval.

## Discussion

While the use of non-barrier contraceptive methods seem to have become a common practice in present day, unprotected sex with the risk of contacting sexually transmitted infections including HIV remain high. Dual protection is protection against unwanted pregnancy, HIV and other sexually transmitted infections and a means of achieving safer sex and birth control [10]. The use of barrier contraceptives like condom provides dual protection. Barrier methods like condoms could serve as contraceptive and barrier to infection in certain circumstances, and as barriers to infection only in others; such as women who have undergone sterilization or who are post menopausal. Dual method is currently being advocated as a cost effective and evidence-based option for HIV infected individuals in achieving dual protection against HIV/STI’s and unplanned pregnancy [1,2]. This present study revealed that HIV infected individuals could be unaware of their HIV status or fail to disclose their status to their sexual partners and still practice unprotected sex with them even when aware. The high fertility, sero-discordant and sero-concordant rates amongst the respondents in this study justifies the need to promote the use of dual method as a form of safer sex practice by all HIV infected individuals as per recommendation [1,2] and also because unsafe sexual interaction between HIV-sero-positive and sero-negative individuals could fuels the HIV/AIDS pandemic which is counterproductive to the global efforts to eliminate new infections by 2015 [3,7]. The findings in this present study were in tandem with the findings of other researchers where one or both sexual partners were found to be unaware of their HIV status and those who were aware were either sero-discordant or sero-concordant [11]. Researchers have reported various sexual practices like heterosexuality, bisexuality and homosexuality as common practices amongst the HIV infected. These were similar to our findings except for homosexuality. These sexual practices were also reported to be influenced by a number of factors such as race, culture, education and beliefs, with an associated increased risk of transmission of HIV and unintended pregnancy [12]. Though heterosexual practice has been implicated in over 80% of HIV transmission in Nigeria, homosexuals and bisexuals are also at increased risk [11]. Therefore, dual methods which protects against STIs/HIV and pregnancy would be beneficial in such setting. The fact that most of the participants were mainly within the reproductive age and sexually active group have great implications for HIV transmission between partners and mother-to-child transmission (MTCT) of the virus, as well as the World Health Organization target of “getting to zero”, reflected by the theme of the last two worlds AIDS day celebration (1st December, 2011 and 2012). Getting to zero aims at zero new infection, zero deaths, zero stigmatization and discrimination [3]. Despite the promotion of dual method as a cost effective and evidence-based option in achieving this target, only few HIV infected women and their sexual partners in the present study were aware of its availability and benefits and also practiced it as a form of safer sex against STIs/HIV and unplanned pregnancy. Dual method could be a veritable tool in sub-Saharan African countries like Nigeria where high fertility rate, heterosexual transmission and unplanned pregnancies have adversely limited efforts in curbing the HIV/AIDS scourge. A third of the respondents were unemployed, while about two-third lived in rural areas with high illiteracy level, these and other factors such as inability of women to negotiate safer sex, poor risk perception, gender economic inequalities and power imbalances have been shown to negatively influenced safer sex practices among women generally [13-16].

Despite the fact that over 70% of the participants had known their HIV status for more than a year, they still demonstrated a high level of unawareness and failed to use dual methods for safer sex, indicating there is an urgent need to rapidly scale up sensitization and uptake as a way of promoting safer sex practices and its benefits among sexually active and/or child bearing HIV infected women and their partners.

More than a tenth of the respondents were involved in multiple sexual relationships within a year preceding the survey and of these, only 17.4% used dual methods against STIs/HIV and unplanned pregnancy, while only about half of them used the condom component only. Considering Nigeria’s high HIV prevalence rate (4.1% in 2010), this risky sexual behavior which is similar to that reported in other regions of the world [1,4] can be a major factor that could further aggravate the current HIV/AIDS situation and thwarts the country’s efforts to achieve the United Nations Millennium Development Goal 6 by 2015. It was noted that even amongst the few users of dual methods, only a mere 26.8% practiced it as a form of safer sex consistently, while majority were inconsistent in its use, this constitutes a major risk factor for STIs/HIV and unplanned pregnancy and may explains the high prevalence of STIs and unplanned pregnancy noted among respondents who were non users or inconsistent users. Similarly, a New York study revealed poor practice of safer sex in heterosexual non-commercial sexual relationships in which only 32% used condoms consistently [17]. The fact that people may fail to practice dual methods/protection consistently and correctly is not a valid reason not to promote its use. It is never too late for those providing family planning and STIs/HIV prevention services to start promoting dual protection with dual methods. In the long-term, the development of highly efficacious and highly acceptable methods of dual protection is an urgent research priority [10]. The findings from this study, emphasizes the need to create awareness, provide and embrace more modern forms of dual methods with higher effectiveness and minimal side effects to improve uptake [18].

The finding that the non practice of safer sex using dual methods was due to lack of awareness, non-disclosure of HIV status, decline by partners, non-availability, religious beliefs, cost, culturally barriers and reduced sexual satisfaction as also reported by similar studies [19], emphasizes the need to step up public enlightenment and the need for disclosure of status. The provision of dual method options should be provider initiated just as advocated and practiced with HIV testing and counseling. Counseling on the benefits of disclosure of one’s HIV status to a sexual partner should be encouraged to improve uptake. Also, modern and effective forms of dual methods should be made readily available and accessible to HIV infected partners at no or minimal cost, cultural and religious barriers that inhibits uptake should be addressed along with the myth or belief that condom use reduces sexual satisfaction, these interventions will go a long way in helping combat the HIV/AIDS scourge, since women generally are not in position to negotiate safer sex by virtue of gender inequality, poor socio-economic status and cultural practices which put them at a disadvantage [20-24].

The main limitation of this study was its small sample size and the fact that it was a hospital based study. A population based study with a larger sample size is therefore recommended for future research.

## Conclusions

The awareness and uptake of dual methods as recommended by the World Health Organization and the Nigeria national guidelines for prevention of mother-to-child transmission of HIV is still very low and thus associated with significantly higher risk of STIs/HIV transmission and unplanned pregnancy. Response to this problem requires massive, multi-sectoral, and well coordinated national and global efforts to implement state-of-the-art interventions to create awareness on the need for and the benefits of using dual methods by all sexually active HIV infected partners as a way of promoting the reproductive rights of women to safer sex and birth control.

## Competing interests

The authors declare that they have no competing interests.

## Authors’ contributions

LOL designed the study, oversaw its conduct, data analysis, and interpretation and drafted the original article and reviewed the final draft. OAK contributed to the design of the study, collection and analysis of the data, and review of the article. IAC assisted in the drafting of the article, data analysis, interpretation and review of the article. All authors read and approved the final draft of the article.

## Pre-publication history

The pre-publication history for this paper can be accessed here:

http://www.biomedcentral.com/1472-6874/14/39/prepub
